# Sources of Tuberculous Infection

**Published:** 1912-08

**Authors:** 


Sources of Tuberculous Infection.—Knopf, (*N. Y. Med. Jour.* June 29, 1912.) estimates that of the 150,000 who die annually from tuberculosis in the United States, 50,000 are bread winners. If a value of only $1500 is set on each life the loss to the community reaches $75,000,000. Another third probably are school children. Making the average duration of life only 7.5 years and figuring the cost to the parents of each child as only $200 per annum, the community loses another $75,000,000.

Children who live in close association with tuberculous parents are found to be tuberculous in 51% of the cases.

Biggs, Brouandel, Naegeli and many others assert that the vast majority of city people living in cramped quarters have or have had tuberculosis. Knopf believes that at least 25% of the children of the masses have an undiscovered tuberculosis. In large families it is nearly always the later born who contract the disease. The age of the father, physical condition of the mother and poverty are all factors.

Direct bacillary transmition through the placenta is rare but occurs perhaps more frequently than our statistics would seem to indicate. However postnatal infection is the most frequent cause. Alcoholism is a strong predisposing cause. There is no doubt that the infection is not infrequently transmitted by the mother's milk. While the bovine type may be considered a negligible factor in pulmonary tuberculosis of the adult, in children it is responsible for a large percentage of cervical, alimentary and bone and joint infections. The next most frequent primary sources of infection in childhood arise from the bacilliferous saliva and sputum of the tuberculous individual in close and prolonged contact with the child.

Among the rarer modes of infection in the adult are mentioned: The tuberculous cigar maker, glass blower, waiter, maid, bookkeeper, kissing of relics among Roman Catholics, inoculation by tattooing and circumcision. Poncet of Paris asserts that he has found the bacillus in the prespiration of consumptives.

As one method of combating the disease Knopf recommends vasectomy in tuberculous cases who insist upon marrying.

